# Clinical Outcomes of a Hybrid Model Approach to Applied Behavioral Analysis Treatment

**DOI:** 10.7759/cureus.36727

**Published:** 2023-03-27

**Authors:** Anurag Garikipati, Madalina Ciobanu, Navan Preet Singh, Gina Barnes, Jenna Decurzio, Qingqing Mao, Ritankar Das

**Affiliations:** 1 Research and Development, Montera, Inc. DBA Forta, San Francisco, USA; 2 Engineering, Montera, Inc. DBA Forta, San Francisco, USA; 3 Executive Leadership, Montera, Inc. DBA Forta, San Francisco, USA

**Keywords:** treatment, patient outcomes, autism, hybrid model aba, applied behavior analysis

## Abstract

Objective

This study examines the implementation of a hybrid applied behavioral analysis (ABA) treatment model to determine its impact on autism spectrum disorder (ASD) patient outcomes.

Methods

Retrospective data were collected for 25 pediatric patients to measure progress before and after the implementation of a hybrid ABA treatment model under which therapists consistently captured session notes electronically regarding goals and patient progress. ABA treatment was streamlined for consistent delivery, with improved software utilization for tracking scheduling and progress. Eleven goals within three domains (behavioral, social, and communication) were examined.

Results

After the implementation of the hybrid model, the goal success rate improved by 9.7% compared to the baseline; 41.8% of goals showed improvement, 38.4% showed a flat trend, and 19.8% showed deterioration. Multiple goals trended upwards in 76% of the patients.

Conclusion

This pilot study demonstrated that enhancing the consistency with which ABA treatment is monitored/delivered can improve patient outcomes as seen through improved attainment of goals.

## Introduction

Applied behavior analysis (ABA) is a treatment that focuses on implementing changes in operant behaviors through varying forms of reinforcement that can help to eliminate or encourage certain behaviors [1,2]. The purpose of ABA is to increase the number of desired behaviors, decrease the number of obstructive and harmful behaviors, and maintain positive and learned skills in a variety of environments. It is an unrestricted treatment that can be adapted to best suit an individual patient's symptoms and is typically associated with treating individuals with autism spectrum disorder (ASD). ASD is a life-long neurodevelopmental disorder that presents heterogeneously among diagnosed patients [3]. ABA treatment relies on positive reinforcement and identifying the chain of events related to a particular behavior. No ABA program is alike, as each patient requires individualized interventions [4].

An ABA program is designed and overseen by a board-certified behavior analyst (BCBA) based on intake forms, patient analysis, and goals set by the patient and their caretakers. The goals within an ABA treatment program individually target deficits related to specific skills. Registered behavior technicians (RBTs) are trained and supervised by BCBAs and work directly with patients to promote and track progress. ABA treatment can be completed in a variety of spaces (e.g., home, school). In fact, holding ABA treatment sessions in varying settings is encouraged to ensure that the patient’s behaviors can be replicated under different circumstances.

ABA interventions are historically effective [5] and have been shown to improve outcomes such as behaviors, feeding disorders, academics, social functioning, independent living skills, and vocational skills for children ranging in age from one year to adolescence that have been diagnosed with ASD [6-9]. However, the traditional ABA model is intrinsically disorganized and lacks a uniform approach to treatment delivery. Inconsistent treatment approaches and delivery result in a lack of availability of broad populations from which treatment responses can be generalized in research [10]. This is despite the fact that the Behavior Analyst Certification Board (BACB) provides guidelines for ABA treatment [4,11,12].

Regarded as the “gold standard” of ASD treatment, research suggests that ABA yields better outcomes than non-ABA or mixed-method therapies [5]. However, treatment methods and targets for ASD are diverse and often include non-ABA techniques or use partial components of ABA, such as the developmental social-pragmatic model [13]. Alternative routes for ASD care may include medication, specific and targeted therapies (e.g., speech, occupational, sensory therapies), cognitive behavioral therapy, or other alternatives presented by healthcare providers [14-18]. These therapies can be used alone or as part of an ABA treatment plan. However, alone, alternative methods are not always supported by sufficient evidence to indicate improved outcomes in ASD patients [17,18].

Technology has been explored for the delivery and improvement of care in ASD treatment and screening/diagnosis [19,20]. Artoni et al. conducted a pilot study assessing the effect of augmenting ABA treatment with a web-based application and showed that 98% of users demonstrated improvement in skills and communication abilities [21]. Simeoli et al. explored the use of a speech-generating tool to improve the communication abilities of an ASD patient and yielded improvement in the patient’s “vocal production” and general communication abilities [22]. Husni et al. examined the use of a mobile application to aid vocabulary development to improve communication and reported that over half (60%) of users strongly agreed that they would recommend the use of the application to supplement vocabulary-building in ABA treatment [23]. Despite this body of research related to implementing technology to improve outcomes and access for patients receiving ABA treatment, its use is not yet widely implemented in ABA practice, whereas its use in other areas of the healthcare sector has been transformative for diagnostic and treatment purposes. The lack of uptake for even basic technology to diagnose ASD and subsequently individualize, deliver, and monitor a consistent approach for ABA therapy is a missed opportunity in which many individuals in need of treatment are left to wait for up to four years for initiation of treatment [24]. These barriers add to the financial, logistical, and intrafamilial struggles that exist for families with children who have ASD. 

Given the complex and heterogeneous nature of ASD and the required individualization of ABA treatment plans, technology can be a powerful tool to provide a more consistent approach for the delivery of ABA treatment and for maximizing desired outcomes in ASD patients. More advanced technology, such as artificial intelligence, may also have the potential to identify individual patient traits and objectively recommend personalized treatment approaches to optimize outcomes [25]. This clinical outcomes paper serves the dual purpose of contributing to the body of research about outcomes related to ABA therapy and discusses how implementing changes to a traditional (or conventional) ABA treatment delivery model (thus resulting in a “hybrid model”) can impact patient outcomes. The term “hybrid” in the context of the model refers to the fact that the hybrid treatment delivery model retains aspects of traditional ABA (such as the content of the delivered ABA treatment) while significantly enhancing consistency aspects of ABA treatment delivery via the implementation of technology (e.g., tech-enabled approaches, such as electronically monitoring, recording, and reporting patient progress, as well as scheduling software) and increased focus on customer satisfaction. To assess the impact of this hybrid model, we examined patient outcomes prior to and subsequent to the implementation of the consistency-enhancing changes to ABA treatment delivery. Under this hybrid model, we improved upon traditional ABA treatment delivery by increasing the use of tech-enabled approaches, which remain underutilized in ABA treatment despite their almost universal presence in other healthcare areas for over a decade. We hypothesized that the hybrid model approach benefits the treatment process for both providers and caregivers, in part by creating more consistent, objective, and structured methods for monitoring and reporting patient progress. We additionally implemented a more robust patient and caregiver (i.e., customer) service approach and hypothesized that improved caregiver satisfaction positively influences patient outcomes. The resulting “hybrid” model, under which we use approaches that are non-standard in ABA (i.e., tech-enabled approaches) and those which should be a component of any healthcare service (customer satisfaction), are presented here, detailing our observations that the hybrid model afforded improvements to the outcomes of ABA treatment.

## Materials and methods

In this retrospective study, the data of patients of Montera, Inc. DBA Forta, San Francisco, United States (hereinafter referred to as Forta) has been used. Collaborative Autism Resources and Education (CARE), LLC was an ABA provider acquired by Forta in March 2022, when the patients of CARE, LLC became the patients of Forta. Following the acquisition, additional tools and processes were implemented while administering ABA treatment starting in April 2022. Patient data were collected from CentralReach (CentralReach, LLC, Fort Lauderdale, Florida, United States), a web-based tool that was used to report, as well as to log and monitor the ABA treatment activity of BCBAs and RBTs. For the entire study period (January-June 2022), the ABA treatment was provided by RBTs under the supervision of BCBAs. The study has been determined to be exempt by Pearl IRB per Food and Drug Administration 21 Code of Federal Regulations (CFR) 56.104 and 45CFR46.104(b)(4) (Protocol #22-MONT-102).

Upon acquisition of CARE, LLC, a hybrid model was deployed for delivering ABA treatment to ASD patients encompassing the following improvements to traditional ABA treatment delivery: (1) ensured technology was widely available to RBTs for the purpose of electronically capturing and reporting session notes (e.g., iPads, laptops, etc.); (2) improved scheduling system of RBT sessions for more efficient delivery of ABA treatment; (3) improved customer service experience for the parents of patients with the top priority of customer satisfaction; and (4) improved internal software and workflow for tracking patient progress. 

**Figure 1:**
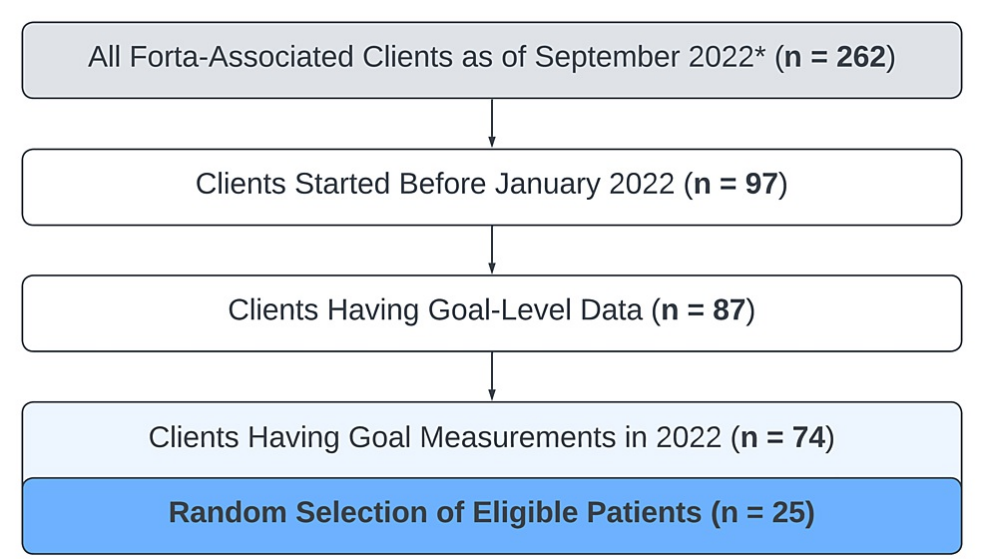
Attrition chart for selection and inclusion of patients. All patients associated with Forta at the time of data retrieval (September 2022) were considered for this study, and the total patient population was filtered down to 74 patients that had all the data necessary for evaluating the potential influence of the hybrid model. From this, 25 patients were randomly selected for in-depth analysis of goal data. ^*Data was extracted and analyzed beginning in September 2022^

The patients for this study were selected in September 2022 from the entire pool of patients (n = 262) having an official ASD diagnosis and associated with Forta (i.e., individuals having records on file) at that time, as shown in Figure 1. The patients were filtered based upon the following criteria: (1) patients (n = 97) began ABA treatment with Forta prior to January 2022; (2) patients (n = 87) had goal-level data available (i.e., quantitative data entered into the CentralReach system to reflect treatment progress); and (3) patients (n = 74) had goals evaluated for overall progress during 2022. Out of 74 patients that met the selection criteria, 25 patients were randomly selected and their data were evaluated for the potential influence of the hybrid model. 

It should be noted that any single patient may have a rather large amount of data collected (sometimes on a daily basis), which amounts to a substantial data volume across the span of six months. There were several limitations on funding and costs of data acquisition/processing due to data being collected through a real clinical practice vs. a curated research dataset. Also, some clinical data were not collected or stored electronically in the time period prior to the acquisition of CARE, LLC by Forta (January-March 2022). Furthermore, data were not always formatted in a manner conducive to statistical research analysis. Consequently, a random subset (n = 25) of the patients that met all selection criteria was chosen for the data analysis presented in this study. Of the patients who met the selection criteria, a random selection of 25 was chosen when the initial data pull was performed. The random selection used a standard random selection algorithm available in SQL database querying to select 25 patients from the database housing CentralReach data. The identification numbers associated with these 25 patients were utilized for collecting all remaining data for each patient and were the basis for the entire analysis.

Subject demographics for the 25 randomly selected patients whose data were subjected to processing and analysis are displayed in Table 1. Overall, the average age was 7.9 years (aged 3.3-15.9 years at the beginning of the study in January 2022), and as expected, there was a greater number of males [11,12]. The patients received a similar weekly ABA treatment in January-March 2022 (average was 19.5 hours of weekly ABA treatment) and in April-June 2022 (average was 19.3 hours of weekly ABA treatment). The average amount of ABA treatment received prior to January 2022 was 20.2 months, ranging from three months to almost 40 months.

**Table 1:** Patient demographic data of sex, age, length of ABA treatment prior to study period, and weekly ABA treatment during January-March 2022 and April-June 2022. ABA: applied behavior analysis

Patient #	Sex	Age in Jan 2022 (years)	ABA length prior to January 2022 (months)	Average number of ABA hours per week	
				January-March 2022	April-June 2022
1	male	7.6	25.5	15.0	15.0
2	female	13.3	25.0	15.0	15.0
3	male	5.3	22.7	18.0	18.0
4	male	4.8	21.3	21.5	21.5
5	male	7.2	18.9	15.0	15.0
6	male	10.7	17.7	19.6	18.3
7	male	4.1	16.2	39.8	37.5
8	male	4.2	13.9	32.5	32.4
9	female	4.0	12.9	27.5	29.1
10	male	6.0	11.8	25.0	25.0
11	male	6.6	8.9	15.0	15.0
12	female	4.4	7.5	25.0	25.0
13	male	5.7	8.3	29.1	28.5
14	female	3.3	6.9	15.0	15.0
15	male	6.4	6.8	12.0	12.0
16	male	8.0	5.1	16.9	17.4
17	male	8.4	4.5	25.0	25.0
18	female	11.8	3.0	34.0	31.6
19	male	9.0	39.6	15.0	15.0
20	male	15.9	39.6	10.3	11.9
21	male	8.9	39.5	6.5	6.0
22	male	8.5	39.6	15.8	15.0
23	male	12.6	39.6	12.0	12.0
24	male	7.0	38.6	20.0	20.0
25	male	15.1	31.6	6.0	6.0

Two types of goals were evaluated: percentage goals and frequency goals. The majority (76.8%) were percentage goals, where the patient was asked to attempt completing a particular task a specific number of times. The value collected for the percentage goals was the percentage of attempts the task was completed successfully during the course of an ABA treatment session. Percentage goals were expressed as a percentage value ranging from 0% (a particular task was never completed) to 100% (a particular task has been completed for the entire specified number of times). For example, if a patient was asked five times to follow a set of three-step instructions and the patient followed that particular set of three-step instructions two times, then the numeric value of the goal percentage was calculated to be 40%. The remaining goals (23.2%) were frequency goals, where a particular behavior was exhibited by the patient over the course of a treatment session. Frequency goals are expressed as a numeric value over time, for example, the number of behavior occurrences (or events) per hour or the number of behavior occurrences (or events) per treatment session. Frequency goals are commonly targeted for reducing undesired behavior (e.g., reducing the number of tantrums or events in a treatment session). The numeric value collected by an RBT is typically the number of occurrences of a particular behavior (i.e., the number of events that are tracked) during a treatment session. For example, if a patient exhibits four tantrums during a treatment session, the frequency goal is expressed as four tantrums (events) per session. 

Percentage goals and frequency goals were evaluated for their status (e.g., improvement or success, setback, lack of change) in the first quarter (Q1) before the acquisition of CARE, LLC (January-March 2022), as well as in the second quarter (Q2) after the acquisition (April-June 2022). Goal data were further evaluated for status change between Q1 and Q2.

In order to compare the goals over time, the mean value of all goals across all patients was computed for each of the six months for which data were retrospectively acquired (January-June 2022). A Q1 baseline value was then calculated as the mean over Q1 (January-March 2022). Baseline values were computed separately for percentage goals and frequency goals. The frequency goals were converted from change in discrete number of events to percent (%) change in events. For each of the six months of retrospective data analysis, a weighted average was computed to account for the percent change of the percentage goals (76.8%) and frequency goals (23.2%). Further, for each of the six months of the analysis, the percentage change from Q1 baseline was computed, highlighting the difference in mean value for each month against the Q1 baseline.

Goals were determined to trend up (positively), trend flat (no significant change), or trend down (negatively), as follows. If a goal trended positively (increase in desired behavior or reduction in undesired behavior), the goal was indicated as trending up. To evaluate the goal trends from Q1 to Q2 (i.e., change in performance from Q1 to Q2), the mean of a particular goal’s value was computed across the entire Q1 and the entire Q2, where the mean for the goal’s value accounted for both percentage goals and frequency goals (i.e., weighted average calculated as a percent (%) value subsequent to converting the frequency goals from change in discrete number of events to percent (%) change in events). Trending up was defined as an increase from Q1 to Q2 in the mean measurement of a percentage goal by at least 5% or a decrease of equal to or greater than one undesirable event in frequency goals (i.e., decreasing from a mean of two undesirable events of hitting to one or fewer undesirable events of hitting). If the behaviors associated with a goal did not change from Q1 to Q2, the goal was indicated as trending flat. It should be noted that the frequency goal data for all 25 randomly selected patients were for reducing undesirable events. Trending flat was defined as the difference in mean measurement of a percentage goal remaining within +/- 5% or +/-1 event in frequency of the mean from Q1. If a goal trended negatively (decrease in desired behavior or rise in undesired behavior), the goal was indicated as trending down. Trending down was defined as a decrease from Q1 to Q2 in the mean measurement of a percentage goal by at least 5% or an increase of equal to or greater than one undesirable event in frequency goals (i.e., increasing from a mean of two undesirable events of hitting to three or more undesirable events of hitting).

## Results

A total of 11 target goals within the domains of behavioral, social, and communication skills were examined during ABA treatment, before and after implementation of the hybrid model (Q1 and Q2, respectively). Within these domains, the targeted goals were intended to address non-compliance, verbal protests, destruction of property, deliberate self-harm, elopement (i.e., leaving a designated area without permission), tantrums, off-task refocusing, staying on task, transitioning from preferred to non-preferred activities, requesting (manding for) a break, and off-task behavior reduction using a five-minute interval. Goal progress over time was determined using the data collected throughout ABA treatment (i.e., Q1 and Q2 data).

**Figure 2:**
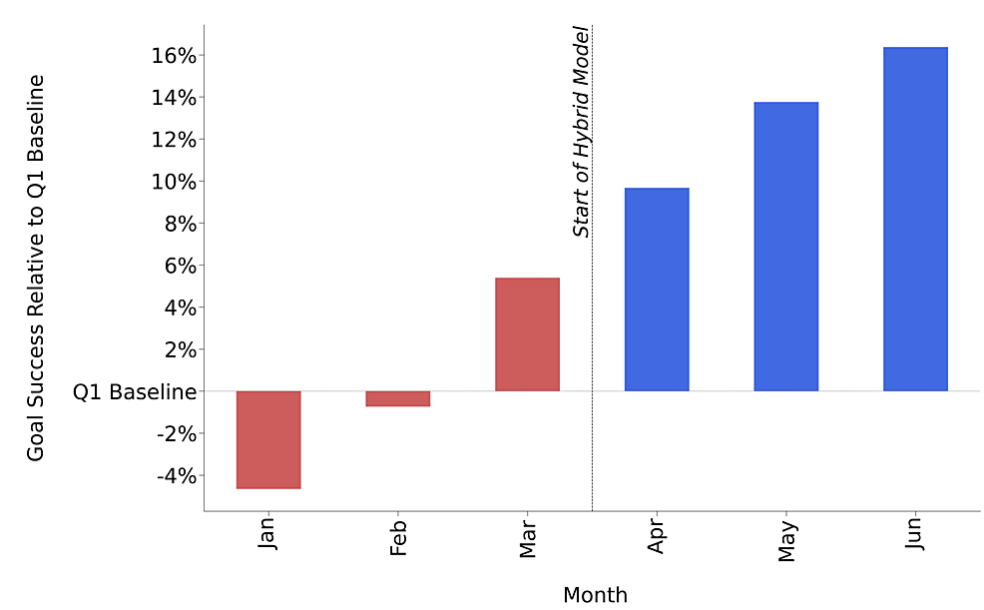
Goals performance before (red) and after (blue) implementation of the hybrid model for delivering ABA treatment to ASD patients. Goal success relative to the Q1 baseline was markedly increased upon implementation of the hybrid model. ABA: applied behavior analysis; ASD: autism spectrum disorder; Q1: quarter 1

To determine the influence of the hybrid model on ABA treatment effectiveness, goal success was evaluated for each month in Q1 and Q2. Figure 2 displays the relative success of goals by comparison to the Q1 baseline for each of the three months in the traditional care model (Q1), as well as each of the three months during which the hybrid model was deployed (Q2).

During Q2, the goal performance of patients improved significantly, with an increase in goal success relative to the Q1 baseline ranging from 9.7% in April to 16.4% in June. Overall, goal improvement was markedly greater upon introduction of the hybrid model for delivering ABA treatment to ASD patients.

**Figure 3:**
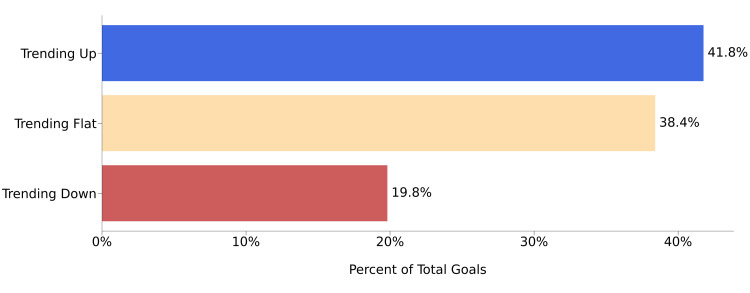
Goal trends subsequent to the implementation of the hybrid model for delivering ABA treatment to ASD patients. The majority of the goals either trended up or did not substantially change in Q2, and less than 20% of the goals trended down. ABA: applied behavior analysis; ASD: autism spectrum disorder; Q2: quarter 2

The influence of the hybrid model on the ABA treatment effectiveness was further assessed by examining the goal trends in Q2 with respect to Q1 (Figure 3). A significant portion of the goals (41.8%) showed improvement, and an almost equal amount (38.4%) displayed a flat trend in Q2, while a minority of the goals (19.8%) indicated deterioration. The data in Figure 3 are averaged over all 25 selected patients and all of their goals.

**Figure 4:**
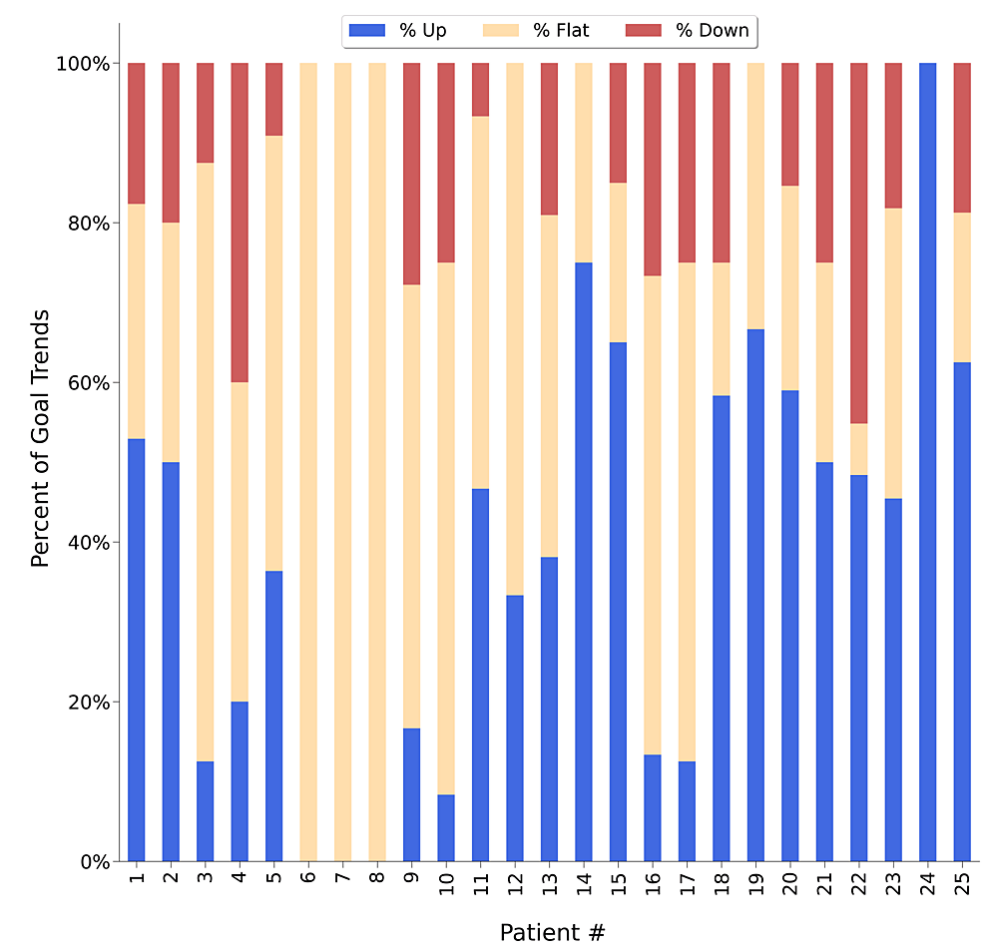
Distribution of goal trends for each patient. Each vertical bar represents a patient’s total progress upon introduction of the hybrid model for delivering ABA treatment. For each patient, blue sections indicate the percentage of goals that trended up, orange sections indicate the percentage of goals that trended flat, and red sections indicate the percentage of goals that trended down. ABA: applied behavior analysis

Out of the 25 patients included in this clinical study, one patient (#24) had all goals trending up during Q2, and there was no patient fully trending down (Figure 4).

Seven patients (#6, #7, #8, #12, #14, #19, and #24) had no downward trend in goals after the implementation of the hybrid model for delivering ABA treatment (Q2). Three patients (#6, #7, and #8) trended flat for all of their goals. The majority of the patients (n = 18) whose data were examined displayed a combination of goals trending up, trending flat, and trending down. Out of the 25 patients randomly selected for this retrospective study, 64% of the patients had the majority of their own goals trending up and 76% had multiple goals trending up. No patient displayed more than half of their goals trending down.

## Discussion

Goal improvement is a fundamental component of the standard of care in ABA treatment, though patient progress is not always measured consistently and may reflect improvement in relatively narrowly-defined areas (e.g., intelligence quotient (IQ)) as opposed to goals that improve overall patient outcomes [26]. This paper provides a retrospective assessment of how the changes we enacted may have impacted skill acquisition as measured by improvements in goal attainment for patients. Subsequent to the acquisition of CARE, LLC, we implemented a hybrid model for the delivery of ABA treatment to ASD patients under which we increased consistency of treatment delivery by integrating streamlined processes and basic but underutilized technology with traditional ABA methods (i.e., while adhering to BCBA recommendations) to monitor and analyze goal (i.e., skill, behavior) improvement.

With regard to process improvements, we placed stronger emphasis on consistency with how treatment was delivered and how progress towards goal attainment was monitored and evaluated, which is a guiding principle of ABA [2]. We employed a streamlined internet-based application for recording and analyzing patient data which required goal progress to be recorded within preset categories (as opposed to allowing progress to be recorded with free text), thus providing a more consistent and objective method of data tracking and analysis. The use of this system had the additional benefit of allowing RBTs to track the number of hours spent with a patient, which ensured that RBTs were continuously aware of the number of remaining hours required to complete treatment towards a particular goal. Further, we ensured broad access to the necessary tools (e.g., iPads, laptops, etc.) for RBTs to capture and deliver session notes electronically. This provided a portable, convenient, reproducible, and reliable mechanism for data collection and for easy visualization of progress in terms of hours logged and successful completion of goals. Though electronic systems are generally available in healthcare settings and practices, such systems were not being consistently used by CARE, LLC at the time of acquisition by Forta and prior to the changes we implemented. To our knowledge, only one other pilot study examined the use of basic electronic tools (e.g., web-based application) to assist with streamlined and consistent delivery and monitoring of ABA treatment within a pediatric population [21]. Therefore, while technology may not be novel within many clinical healthcare settings, the lack of research examining such an approach for ABA treatment delivery indicates a gap in research.

Under the hybrid model, we observed consistent improvements in goal achievements by comparison to the baseline measurements taken prior to the implementation of the hybrid model (Figure 2). Overall, the patient population had 41.8% of goals trending up and 38.4% of goals trending flat (Figure 3). This may be indicative of the hybrid model having a positive influence on the progression of ABA treatment. A case study conducted by Meçe and Sherifi examined ABA treatment outcomes in a similar manner (i.e., reflected by growth in goal attainment) [27]. In their study, to ascertain the patient’s progress, 13 goals/skills were assessed between the baseline and study conclusion to determine the level of completion within each domain. Their patient advanced in all domains and growth of goals demonstrated a broad range of success (4-40% per year). When broken down for individual patients, in our study, we observed that a majority of goals trended upward or flat following the implementation of the hybrid model (Figure 4), which may indicate that progression through the ABA treatment program was improved upon the introduction of the hybrid model. When goal trends were observed individually for each patient following the introduction of the hybrid model, a significantly smaller proportion of our patients regressed in goal attainment. There are many potential reasons for this regression. However, regression during the course of ABA treatment is a known phenomenon [6,27,28]. Research has frequently explored inconsistency within ABA treatment response, even in instances where patients are receiving the same treatment intensity and duration [6,28]. Factors that may impact treatment response are numerous and may include traits of the individual patient (demographics, specific characteristics of the patient’s ASD, IQ), baseline language abilities), treatment dosage, and targeting treatment goals that require a longer duration or higher intensity of treatment to master [6,28,29]. As noted by Choi et al., individual patient progress may also fluctuate over time due to goals being more finely tailored for the patient’s skill level and the likelihood of achieving a goal in a particular domain [26].

Examining the distribution of goal trends for each patient (Figure 4) in the context of the patient demographics (Table 1) revealed no apparent pattern, indicating that the consistent improvements in goal achievement by comparison to the baseline (Figure 2) were largely due to the implementation of the hybrid model during Q2. The ABA treatment intensity remained substantially the same for all patients between Q1 and Q2, with few exceptions, and the magnitude of the difference in the number of ABA treatment hours per week between Q1 and Q2 was not significant (19.5 hours/week and 19.3 hours/week, respectively). Further, the ABA treatment intensity did not appear to influence the trend of the goal progress. However, nine patients had received ABA treatment for at least two years by January 2022 (#1, #2, #19, #20, #21, #22, #23, #24, #25), and they appeared to be more susceptible to the benefits of the hybrid model. These nine patients had a higher proportion of goals showing improvement in Q2 when compared to the average number for all 25 patients (59.4% vs. 41.8%), which in turn resulted in a decreased amount of goals displaying a flat trend (22.8% vs. 38.4%, respectively) or deterioration (17.8% vs. 19.8%, respectively) in Q2 when compared to the average number for all 25 patients. While research suggests that improvements in adaptive behavior can occur at a slower rate after two years of ABA treatment, it is also noted that using a tailored and patient-centered approach may continue to yield gains toward goal attainment even after two years of ABA treatment [26]. Our hybrid model for ABA treatment delivery enhances the consistency with which ABA treatment is monitored/delivered, which in turn can improve patient outcomes as seen through improved attainment of goals (for some patients even after more than two years of ABA treatment). This appears to be in agreement with the findings of Choi et al.

Initiating and validating a hybrid model for ABA treatment delivery to ASD patients can prove to be advantageous for both treatment providers and patients. First, it can be logistically challenging to locate ABA providers due to shortages of certified individuals that have the specialized training required to deliver ABA treatment [30]; there can also be challenges finding ABA providers in the vicinity of a patient’s home, making it difficult for parents and caregivers to attend treatment sessions with the frequency needed to improve goals [4]. In our hybrid model, we reduced these challenges by improving scheduling and providing more readily available customer service to the patients’ caregivers to enhance effective treatment delivery. As facilitating access to care may increase an individual’s likelihood of pursuing treatment, and thus improve patient outcomes [31,32], it is possible that these changes contributed to the goal-level improvements that were observed. We additionally had the BCBAs monitor treatment remotely, which may have minimized logistical barriers associated with BCBA shortages [30]. Improved outcomes may also be due to the RBTs electronically capturing and reporting session notes.

Limitations and future work

It is important to acknowledge the limitations of this retrospective clinical study. First, it was a pilot study with a limited sample size; therefore, the results are not generalizable. Future studies should examine trends in similar goal attainment in a larger patient sample to better understand the influence of non-conventional approaches to ABA treatment (one example of which is our hybrid model for delivering ABA treatment). Second, the study only covers six months, and it is possible that alternative patterns could have emerged over a longer time period. Future studies may take a longitudinal approach to examine outcomes in order to examine patterns in patient outcomes over time. We were unable to determine specific factors that contributed to patient outcomes in our retrospective study, such as individual traits (i.e., skill levels towards accomplishing various tasks, IQ, etc.). This made it difficult to understand what degree any external influences have on ABA treatment compared to specifically implemented changes to treatment delivery [6,28]. Future studies may include attempting to identify patterns in which particular goals take longer to master and traits of the hybrid ABA treatment that contribute to higher growth of goal attainment. The reported results are based on retrospective data. While the nature of the data analysis (i.e., pre- and post-hybrid model implementation) renders the current study a proxy for quality improvement (QI) research, future work may encompass a prospective study. Lastly, the technology implemented in the study was routinely available, though not broadly in use for managing ABA processes. Future studies may involve the use of more advanced technology (i.e., machine learning) for determining the intensity level and focus areas within therapy to deliver to individual clients based on individual traits as well as for analysis of outcomes. Such studies could determine the influence that more advanced technology and personalization of treatment approaches could influence patient outcomes.

Although the modifications that we made to treatment delivery through the implementation of the hybrid model mitigated to a certain extent (as evidenced by the patient outcomes data presented in this paper) some of the intrinsic inconsistencies of traditional/conventional ABA treatment delivery approaches, this hybrid model does not fully solve the lack of consistency in ABA treatment delivery or the issue of BCBA and RBT shortages. Subsequent to the deployment of the hybrid model in Q2, we expanded the scope of the ABA treatment delivery framework to allow parents of patients with ASD to complete the appropriate training and deliver parent-led ABA treatment sessions. There is ample evidence that ABA treatment delivered by parents can achieve similar outcomes to ABA treatment delivered by trained therapists [15]. The former method of treatment delivery can display increased consistency, for example by eliminating the change in therapists that can occur in traditional/conventional treatment delivery approaches. Further, research has suggested that parents can successfully increase their knowledge regarding ABA and effectively deliver treatment, and that the greater degree of flexibility to select a convenient time for treatment may positively influence the amount of time that a parent can devote to ABA [33], which in turn can improve treatment consistency and quality. In a preliminary analysis of an example of parent-led ABA treatment, a patient (aged 10 years, female, with an official diagnosis of ASD) showcased elevated levels of progress on eight goals within one month of beginning treatment, with 50% of the goals showing substantial improvement (trending up) in subsequent months, when compared to the first month. Similarly, an additional 25% of goals showed a marginal improvement (marginally trending up), while 12.5% of goals remained consistent (trending flat). Only one goal trended down for the patient and the goal only showed a slight decrease in performance. The preliminary analysis of the parent-led treatment sessions indicates an improvement in goal performance. It should be noted that regardless of whether the treatment was delivered by an RBT or was delivered via parent-led ABA treatment sessions, progress was monitored virtually by a BCBA, per BACB guidelines [4,30]. Future work will involve analysis of the patient data to determine if the hybrid model leads to better outcomes when ABA treatment is delivered by an RBT, or when ABA treatment is delivered via parent-led sessions.

## Conclusions

While ABA is the gold standard of ASD treatment, implementation is often inconsistent and patient outcomes are highly heterogeneous. Despite this, increasing the consistency, organizational structure of delivery methods, and access to care using a hybrid model demonstrated improved outcomes for the ASD patients in this study. By combining a variety of tools (e.g., wide access to electronic monitoring, recording, and reporting of patient progress and improved scheduling, customer service, and customer tracking software), the overall efficiency of ABA treatment was increased, which was reflected in better patient outcomes, as evidenced by the significant improvement in goal performance. Such a hybrid model may hold promise for providing broader access to ABA treatment, improved consistency in the approach to care delivery, and routinely improved outcomes for patients with ASD.
